# Is a European Recovery Possible Without High-Tech Public Corporations?

**DOI:** 10.1007/s10272-021-0973-x

**Published:** 2021-06-04

**Authors:** Daniele Archibugi, Vitantonio Mariella

**Affiliations:** Institute for Research on Population and Social Policies, Italian National Research Council, Via Palestro, 32, 00185 Rome, Italy

## Abstract

Pervasive new technologies associated with information and communication technologies and software are dominated by a restricted oligopoly of US-based corporations. The challengers are no longer European firms, but rather Japanese or Chinese companies. The actions taken by the EU to fill this technology gap, including the Framework Programmes for research and technological development, are beneficial but still insufficient in terms of the resources committed. This article argues that the EU urgently needs to add another economic policy instrument to defy these incumbent firms, namely to create a few publicly supported large corporations in the areas of greater scientific and technological opportunities. This will be complementary to the already ongoing mission-oriented innovation policies. While there are the political and economic difficulties of implementing such a strategy, one recalls the pioneering venture of Airbus, established more than 50 years ago that has successfully managed to challenge the dominant US-based passenger aircraft producers despite several economic and political controversies. Could similar attempts be replicated for green technologies, healthcare services and artificial intelligence?

There is a consensus that Europe will start a solid recovery after the COVID-19 crisis only if supported by remarkable direct government intervention. The existing policy instruments at the national and European levels, and most notably those made available with the Recovery Fund, support and boost economic, technological, social and cultural development.

## Can the European economic recovery be knowledge-intensive?

One of the key priorities aimed at enhancing the European economy is that of bridging the scientific and technological gap of the EU vis-à-vis the United States and Japan, as these competencies are needed to sustain rising industries. We know that the EU is composed of very heterogeneous countries; while research and development (R&D) intensity, i.e. R&D expenditure as a percentage of GDP, is high in some member states, others are lagging. Overall, the EU has a lower R&D intensity than the US and Japan and it is now challenged by emerging countries such as China (see Figure 1).

For several decades, the EU has carried out a battery of actions to enhance education, science, technology and innovation. Specifically, the EU Framework Programmes started in 1984 and tried to foster European capabilities in promising technological areas. Among them, a crucial role has been devoted to supporting information and communication technology (ICT) clusters, perhaps because they were considered an enabling technology on which the overall economic prosperity depended. How-

* Preliminary versions of this paper were presented at the webinars organised by the Birkbeck College's Centre for Innovation Management Research, University of London, 20 May 2020 and at the Forum "The New European Industrial Strategy after The Great Financial Crisis and the Covid Crisis", 4 February 2021. We wish to thank the participants and Andrea Filippetti, Andrea Guido, Margarita Estevez-Abe and Carlo Milana, for their comments. Grants from the School of Business, Economics and Informatics of Birkbeck, University of London, and the financial support of PRIN (Projects of National Interests promoted by the Italian Ministry for University and Research) Innovation and Global Challenges Prot. 20177J2LS9 are gratefully acknowledged. Usual disclaimers apply.

**Daniele Archibugi**, IRPPS – Italian National Research Council, Rome, Italy; and Birkbeck, University of London, UK.

**Vitantonio Mariella**, IRPPS – Italian National Research Council, Rome, Italy.

Figure 1

**R&D intensity and gross domestic expenditure in China, Japan, the EU and the US, 2000-2018**

percentage of GDP; and total amount in billions of US dollars (size of the circles)

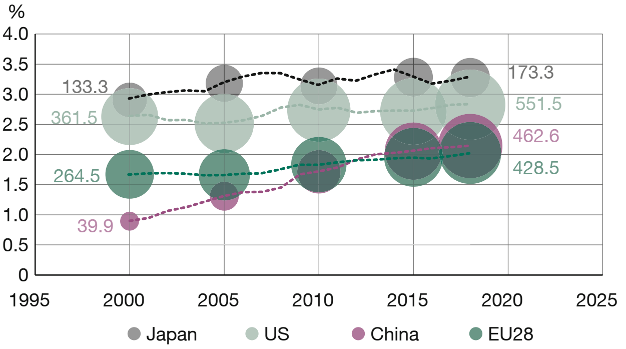

Source: Elaboration on OECD, Main Science and Technology Indicators, 2020.

ever, the gap with the US is still substantial. The Framework Programmes have played a crucial role in creating capabilities across the old continent, also allowing integration and intra-European collaboration among firms and universities, but they have not managed to close the gap, nor could they have achieved such a demanding task on their own.

The overall economic consequences of the 2008 financial crisis have also affected science and technology. The EU level of investment – one of the main engines of innovation – was still below its 2008 level when the COVID-19 crisis broke out. In many EU member countries, public investment, rather than acting anti-cyclically, decreased even more than the business investment. The EU tried to sustain the total level of investment with the European Fund for Strategic Investments, but this also proved to be insufficient (Archibugi et al., 2020).

The current and post-COVID-19 instruments, including the European Recovery Fund, will eventually provide massive resources to support public investment plans and a substantial part will be devoted to R&D and innovation. But the bulk of these resources will be managed by national authorities under European Commission supervision and not, like the Framework Programmes, directly by the European Commission.

This article asks the question: Can the EU fill the technology gap through public investments and incentives to R&D and innovation without also attempting to create enterprises in high-tech industries? We doubt it. Our view is that the interventions aimed at financing and supporting the activities of the existing institutions and firms are certainly useful, yet this may not be enough. We suggest that a cluster of new firms that are able to contribute to the generation of technological opportunities and, above all, the capacity to transform them into viable commercial products, processes and services, may be needed.

To prove our point, a comparison with China is certainly instructive. China has substantially increased the resources devoted to education, R&D and innovation; but to exploit this investment economically, it is bolstering new companies able to compete with big tech American corporations, especially in new strategic industries. In comparison, the EU response is much feebler.

The following section briefly outlines the EU strategies and efforts aimed at enhancing technological capabilities. The possibilities for Europe to set up large public corporations to enable technologies are then explored, including two previous experiences, the Concorde and the Airbus cases. Finally, the article explains how to identify the most promising emerging sectors, with a special focus on the European Battery Alliance.

## The EU regional dimension in scientific and technological capabilities

The EU's problem is that it is a highly heterogeneous area. It differentiates from the US or China in that it does not have the powerful governance devices that characterise nation states yet. The various Framework Programmes were forced to balance two opposite objectives. On the one hand, their goal was to enhance the scientific and technological competencies of the core areas to support European industry's competitiveness against foreign countries. On the other hand, they aimed to foster the development of competencies in the catching-up areas.

Regional imbalances in technological capabilities in the EU are very severe. Whereas some timid signs of convergence have occurred because of the Framework Programmes, regions' contributions to the overall generation of new knowledge are very asymmetric (Archibugi et al., 2021). Eastern European countries, despite their attempt to better integrate into the overall EU scientific and technological communities, have registered small signs of progress in enhancing their innovative capacity. This indicates that the transition from a planned to a market economy has been harder than expected, especially concerning technological developments. Southern European regions continue to lag behind the Northern European countries and have accumulated increased delays in the aftermath of the 2008 crisis.

Furthermore, it has been demonstrated that having a strong, influential network position in collaborative EU

Table 1
**Top corporations' R&D expenditure in 2018 compared to Horizon 2020 average budget**

| Rank | Company | Country | Industry | R&D expenditure (€ billion) | Employees (thousand) | Market cap (€ billion) |
|---|---|---|---|---|---|---|
| 1 | Amazon | | General retailers | 21.20 | 647.50 | 773.52 |
| 2 | Alphabet | US | Software and computer services | 18.27 | 98.77 | 321.57 |
| 3 | Samsung Electronics | South Korea | Electronic and electrical equipment | 14.83 | 309.63 | 243.46 |
| 4 | Microsoft | US | Software and computer services | 14.74 | 144.00 | 752.29 |
| 5 | Volkswagen | Germany | Automobiles and parts | 13.64 | 664.50 | 40.81 |
| | Horizon 2020 | EU | | 13.26 | | |
| 6 | Huawei Investment & Holding Co. | China | Technology hardware and equipment | 12.74 | 188.00 | |
| 7 | Apple | US | Technology hardware and equipment | 12.43 | 132.00 | 960.21 |
| 8 | Intel | US | Technology hardware and equipment | 11.83 | 107.40 | 195 |
| 9 | Roche | Switzerland | Pharmaceuticals and biotechnology | 9.80 | 94.44 | 150.05 |
| 10 | Johnson & Johnson | US | Pharmaceuticals and biotechnology | 9.41 | 135.10 | 315.58 |

Sources: Elaborations on the EU Industrial R&D Investment Scoreboard (2019) and EU Expenditure and Revenue 2014-2020. For Amazon, we use the data provided by Skillicorn (2020).

research greatly affects participation in Horizon 2020 projects (Enger, 2018). The presence of these "closed clubs" has often been at the expense of the less influential higher education institutions located predominantly in the periphery of Europe, leading to a vicious spiral in which established institutions have acquired more funds and reinforced their position.

The EU has a tough choice to make. On the one hand, it should foster EU scientific excellence and technological capabilities vis-à-vis a fiercer global competition with established countries like the US and Japan and emerging countries like China and India. On the other hand, it should also increase EU cohesion by reducing technological disparities across its regions and industries. The two objectives are somehow in conflict with each other. While the former may require a further concentration of competencies in the most emancipated areas to compete with leading technological hubs such as Silicon Valley, Route 128, Samsung town or Shenzhen, the latter may nurture capabilities of the least developed regions and sectors.

What are the instruments available at the EU level? One of the most relevant is certainly the Framework Programmes and it is very likely that the coming Horizon Europe (2021-27) will have to ponder two choices:

- reduce disparities by fostering the distribution of knowledge in peripheral areas and comparatively weaker sectors
- challenge the dominance of the US and China by enhancing the excellence of selected players and areas.

The Horizon 2020 project, which just ended, was one of the world's largest public schemes supporting new knowledge development. Despite the massive resources made available by the EU to enhance scientific and technological capabilities, especially in enabling technologies, they merely corresponded to the yearly equivalent budget of the R&D investment of large corporations. While the Horizon 2020 yearly budget was about €13.2 billion, large corporations such as Amazon (€21.2 billion), Alphabet (€18.3 billion), Samsung (€14.8 billion), Microsoft (€14.7 billion), Volkswagen (€13.6 billion) or Huawei (€12.7 billion) alone spend more or comparable amounts (see Table 1).

Horizon Europe is an excellent financial instrument to generate and disseminate competencies across the EU, however, it will not be able to single-handedly create a genuine industrial capacity to allow the EU to be a world-leading player in emerging technologies.

## Can the EU set up large corporations in enabling technologies?

There is widespread consensus that the state should be a vigilant referee of the competitive process through regulations and antitrust policies. In contrast, there is much more debate on its role as a direct economic player in a market economy. A daring perspective is that European governments should actively participate in the decisions concerning industrial policy strategies, rather than simply act as a regulator (see for example Cimoli et al. (2015) and the other contributors to the same *Intereconomics* Forum). There are several industrial policies that governments carry out to reinforce the presence in innovative industries (Edler and Fagerberg, 2017). But the EU as a whole, with the support of national governments, should attempt to add another economic policy instrument, namely the generation of new firms in the emerging and enabling technologies.

“National champions”, i.e. large corporations able to compete in the global markets, need the support of a proper national government to survive (Strange, 1991), especially if they are associated with complex knowledge infrastructures (Mazzucato, 2013). But fresh national champions would have insufficient strength to compete with the incumbent American and Chinese corporations, particularly because they may receive political protection from the government of their country only. Fast-growing European companies and start-ups, especially in the ICT and related sectors, could easily be acquired by the biggest companies in terms of market capitalisation (market value) and liquid assets (see Rikap and Lundvall, 2020). American Big Tech have already acquired promising European start-ups, a strategy that is widely used to obtain quick and easy access to new technologies and retain market dominance (Marks, 2017). If new start-ups are acquired by foreign big-tech firms, they will indirectly provide public support for the technological advancement of foreign competitors. As shown in Table 1, none of the largest spenders on R&D with gigantic market capitalisation are based in Europe.

The policy implication is quite straightforward: To become a challenger in high technology, we need new publicly supported corporations at the continental level. Have European countries ever joined forces to create companies able to enter new industries and compete with the US? Rarely, but there are two important cases to recall: Concorde, which started as a French-British venture in 1969, and Airbus, which began as a French-German venture also in 1969.

## Lessons from the past: Concorde and Airbus

In the 1960s, European governments decided to produce airplanes as a third player to challenge their two dominant rivals, the US and the Soviet Union. The two superpowers developed competitive airplanes for military purposes and subsequently adopted them to civilian transportation. Since European countries were no longer military leaders, they lacked this capacity. The vulnerability of the European industry, specifically in aircraft, created the political environment to build up such new ventures. Many commentators believed that without them, Western Europe would have been marginalised in the international division of labour (see, for instance, the influential book by Servan-Schreiber, 1968).

The launch of the Concorde by Aerospatiale (France) and BAe (UK) – a jet engine passenger aircraft developed during the 1960s and introduced in 1969 – was one of the first-ever collaborations within the European context, even if it took place outside the institutions of the European Economic Community (in 1969 the UK was not yet a member of the European Economic Community). The Concorde was born because the French and British empires joined their forces to compete with the Soviet Tupolev Tu-144 to produce supersonic transport aircraft. This collaboration is indeed an example of a combination of two existing national trajectories. At the time, France was specialised in jet technology (for military purposes) and the UK had a long record in the passenger market.

While Concorde was a technological success, it ended up being an economic failure. Only 20 airplanes were manufactured, seven of which were acquired by British Airways and seven by Air France, the respective flag carrier airlines. Although the product was well designed and prestigious, it turned out to be a commercial fiasco, mainly due to its impressive consumption and maintenance costs.

The second example is the European Airbus consortium, which started developing aircraft in the 1970s. Airbus has been economically successful and, after half a century, has managed to create a dominant European firm in the industry. Set up as a French-German venture in 1969, Airbus rapidly became a transnational consortium involving Aerospatiale and BAe, the German firm DASA and the Spanish firm CASA. Even this venture developed outside the institutions of the European Economic Community. Its success has paved the way for new European networks, such as Avions de Transport Regional, and recently Aero International Regional.[1]

Airbus challenged the American incumbent airplane manufacturers, all subsidised for military purposes (Boeing, Lockheed and McDonnell Douglas). Similarly, European governments responded with subsidies for R&D, fiscal in-

1 In military aircraft, European collaborations date back to Panavia, established in 1969, and extended to Eurofighter and Europatrol. Similarly, European Helicopter Industry and Eurocopter have become prominent leaders in the European helicopter industry.

centives and political support to urge airline companies to purchase from Airbus rather than US producers. This led to a fierce Atlantic commercial rivalry between the European Union and the US as the governments of each side supported their companies.

Airbus's rivalry with Boeing and McDonnell Douglas led to intense debates in the GATT about the role of public funding in generating "unfair" competition. These cases were later discussed at the WTO, with the US government complaining about the European R&D subsidy to Airbus and the EU equally upset about the US military procurement to Boeing. Eventually, focusing on the civilian component, Airbus managed to generate and maintain cheaper and more consumer-friendly airplanes. In 1994, Airbus sold more commercial aircraft than Boeing for the first time and in 2016 became the first in the world in the sector. Without Airbus, currently, the world market in civil airplanes would be a monopoly in the hands of a single US corporation, Boeing.

Aviation has witnessed a rapid acceleration in transnational networks among firms developing high-risk innovations, and other knowledge-intensive industries have followed the same route. The question here is why countries ought to collaborate. From an evolutionary perspective, one expects that countries in cross-border collaborations recombine their national specialisation pattern. To the extent that two countries are specialised in different technology/market combinations globally, they can collaborate in two ways. Either they recombine the technology in which they are specialised with the market in which the other country is specialised or vice versa. The recombination of specialisation patterns allows partners to explore new technology/market trajectories collectively.

When Airbus began, France had just switched its technological base from jets to turbofans, while the UK was already specialised in passenger aircraft (Frenken, 2000). Hence, previous patterns of expertise reflect the techno-economic specialisation of the transnational network. Germany, however, had lost its expertise in aircraft after WWII, and Spain had little experience. For these countries, Airbus provided an opportunity to leave their old specialisation pattern and enter a new market segment using state-of-the-art technology. Airbus's entry into the aircraft passenger market may be conceived respectively as a reshuffle of competencies for some countries and a developing strategy for others. Overall, governments provided the political support, the financial resources and the expertise, but without a company, it would have been impossible to enter into such a complex and protected market. This demonstrates that when entrepreneurs are not willing to bear risks, the government should intervene directly.

While there was an initial underestimation of the benefits of Airbus's entry into the aviation market (Neven and Seabright, 1995), after half a century it can be considered a vital political and economic choice that produced benefits not only for Europe, but for the whole world – the US included. A new venture in a fast-growing industry prevented the sector from becoming a worldwide monopoly.

## Choosing the new emerging industries

The current American-dominated oligopoly in ICTs bears a strong resemblance to the situation of commercial aircraft in the 1960s. But ICTs today are much more relevant for current and future economic development. Not only are nations that depend on foreign corporations in strategic areas such as communications, satellites, data, social networks and artificial intelligence more vulnerable, but they also lose their technological sovereignty (Edler et al., 2020).

It is certainly not easy to identify the crucial sectors which will be indispensable for future economic, social and political life. One may wonder why shoes and champagne are less relevant than satellites and vaccines, provided that the former are as lucrative as the others. And the fact that the EU has a persistent commercial surplus with the US, even though there are no Big Tech companies located in Europe, may negate the urgency to enter these high-tech sectors. Some sectors, however, are likely to play a paramount role in future economic competitiveness.

There are many ways in which economists can contribute to identifying the strategic industries of the future. The first is to consider the growth rate of production and productivity. But when statistics show that production starts increasing exponentially, the position of nations in the international division of labour has already been established and it is difficult to revert it. For this reason, one may need to use indicators that anticipate upcoming scientific and technological opportunities. By looking at the degree of dynamism and the level of pervasiveness of scientific and technological sectors, it is possible to anticipate which industries will be dominant in the future. The rapidly growing academic literature and patents often indicate the most rewarding scientific and technological areas (Meliciani, 2001). The level of pervasiveness – defined by the variety of users across industries – indicates those enabling technologies that will be necessary for the delivery of most products, processes and services (Evangelista et al., 2018). These areas are likely to have innovations that lead to organisational and social changes to the extent that they can be seen as the backbones of a new techno-economic paradigm (Freeman and Louçâ, 2001).

Policymakers do not necessarily wait for experts' recommendations to decide where to invest. It is self-evident that in crucial areas, such as computers and smartphones, the market share of EU corporations is tiny. EU citizens rely on American social networks, while European institutions have serious difficulties obtaining regulations to protect their data and ensure that proper tax is paid. While China has succeeded in entering new lucrative fields such as smartphones with Huawei and social networks with Tik Toc, the EU has lost its competitive companies (such as Olivetti for computers or Nokia for cell phones) and not even tried to enter into the market of social networks. Similar problems apply for e-commerce: Amazon dominates the European market without being challenged, while China has maintained at least its internal market through Ali Baba. In new enabling sectors like artificial intelligence, the EU investment rate is much below not only that of the US but also that of Japan and China, and, above all, it does not seem that there will be an EU company to gain prominence in the near future (Zachary et al., 2020).

We are not arguing that generating new continental public corporations should be the only industrial policy response to affirm the EU presence in the world economy. In other cases, different attempts could be more fruitful to generate successful industrial capacity in emerging areas (for an overview, see Edler and Fagerberg, 2017). A case in point is the timely venture of the European Battery Alliance.

### Capacity building in an extended industrial network: The case of the European Battery Alliance

> In Europe, within this decade, where it is technologically and economically viable, everything that can be electrified will be electrified, thus making battery technology one of the most important key enablers for the green energy transition facilitating existing and new technologies. (European Commision, 2020, 6)

It is difficult to disagree with such a statement, especially since the European Commission's target is to achieve a successful transition to a fossil-free society, as contemplated by the Green Deal.

In 2017, the European Commission launched the European Battery Alliance (EBA) in the spirit of one of its mission-oriented public programmes (Kattel and Mazzucato, 2018; Mazzucato, 2018, 2019).[2] Industrial alliances allow the facilitation of tighter cooperation and joint action among interested actors, bringing together a wide array of players in a given industry or value chain, including public and private players and civil society. The battery industry does not necessarily require large producers. The common knowledge base is applied to very different products and markets that include specialised operators, general-purpose users and consumers. To catch up, a laggard economic area should carry out a variety of actions; the EU has used several integrated instruments to develop prominence in this specific industry.

The first EU decision in this area is allowing national governments to provide up to €2.9 billion in state aid. Like any custom union, EU institutions are mandated to prevent member state aid that could alter competition. But when state aid is directed towards capacity building, especially in emerging areas in which the EU is lagging behind its competitors, the resources provided by national authorities could be advantageous to all members, and they deserve benevolent consideration.

The second decision is to promote the widespread collaboration and dissemination of knowledge generated across a wide range of players across countries. This was carried out by fostering cooperation and also by dedicating targeted resources within the Horizon 2020 scheme "Next-generation batteries" and similar actions contemplated in Horizon Europe. These ventures will, at the same time, contribute to both collaborative research and innovation ventures as well as the dissemination of knowledge across players.

The third decision focuses on providing loans at negligible interest rates for the battery value chain ventures through the European Investment Bank (EIB). Since 2010, battery projects financed by the EIB totalled €950 million and fostered €4.7 billion of overall project costs. The EIB involvement has significantly stepped up the financing of all the battery value chain stages, ranging from R&D, raw material extraction and processing to battery production, e-charging infrastructure and recycling.

The combination of grants, collaborative ventures, advantageous loans and regulations, together with the commitment to support the industry for several years, will hopefully make the EU a world leader in batteries. But

2 The other EU mission-oriented public programmes are the European Raw Materials Alliance, European Clean Hydrogen Alliance and Circular Plastics Alliance.

such a strategy could be less effective when there is the need to affirm a remarkable fresh presence in restricted oligopolistic markets. In such cases, if the EU wishes to enter into the market dominated by US Big Tech, a more active role is needed, namely the creation of European public corporations.

## The need to add another arrow to EU economic policy instruments

The exogenous crisis represented by COVID-19 will certainly accelerate the global productive organisation. The EU risks falling behind unless its economic activities are adequately supported by government intervention and steered towards the emerging sectors. Horizon Europe will continue to be a crucial policy instrument both to enhance scientific and technological capabilities and to facilitate their dissemination across a rather heterogeneous economic fabric, going from Lisbon to Tallinn. But the Horizon Europe budget is comparable to one of the top high-tech corporations and cannot alone change the landscape.

The massive resources made available through the Recovery Fund are needed to sustain the long-term drop in investments in the EU, which has been especially detrimental for the innovative component. These resources will be administered by national authorities under the European Commission's supervision. However, it is less likely that they will lead to large-scale intra-European technological projects.

Other industrial policy instruments are needed. We have suggested the launch of proper continental public corporations replicating what has been done with Airbus more than half a century ago. It is not difficult to identify those areas where there are greater scientific and technological opportunities and where the EU has either an advantage, such as green technologies and healthcare services, or where it is lagging behind and a gap needs to be filled with the incumbent and challenging nations, such as ICTs and artificial intelligence. These are the areas where genuine European champions could hopefully sustain a solid continental economic recovery.

Although the endorsement of the European Council is certainly needed, these ventures could be initially pioneered by some governments only, in the hope that with time all EU members will join them. They will require building competencies, patient money, entrepreneurship and leadership. These are all resources that are available in the EU that will need to be channelled in new daring routes.
